# Emergency Percutaneous Coronary Intervention Through the Left Radial Artery is Associated with Less Vascular Complications than Emergency Percutaneous Coronary Intervention Through the Femoral Artery

**DOI:** 10.6061/clinics/2017(01)01

**Published:** 2017-01

**Authors:** Guoqing Qi, Qi Sun, Yue Xia, Liye Wei

**Affiliations:** I The First Hospital of Hebei Medical University, Department of Cardiology, China; II The Military General Hospital of Beijing PLA, Department of Cardiology, China

**Keywords:** Left Radial Artery, Femoral Artery, Emergency PCI

## Abstract

**OBJECTIVE:**

To compare the advantages and disadvantages of emergency percutaneous coronary intervention through the left radial artery with those of emergency percutaneous coronary intervention through the femoral artery.

**METHODS:**

A total of 206 patients with acute myocardial infarction who required emergency percutaneous coronary intervention and were admitted to our hospital between January 2011 and August 2013 were divided into the following two groups: a group that underwent percutaneous coronary intervention through the left radial artery and a group that underwent percutaneous coronary intervention through the femoral artery. The times required for angiographic catheter and guiding catheter placement, the success rate of the procedure and the incidence of vascular complications in the two groups were observed.

**RESULTS:**

There was no significant difference in catheter placement time or the ultimate success rate of the procedure between the two groups. However, the left radial artery group showed a significantly lower incidence of vascular complications than the femoral artery group (*p*<0.05).

**CONCLUSION:**

Emergency percutaneous coronary intervention through the left radial artery is associated with less vascular complications than emergency percutaneous coronary intervention through the femoral artery and is thus potentially advantageous for patients.

## INTRODUCTION

Percutaneous coronary intervention (PCI) through the right radial artery has become the first choice for the vast majority of physicians performing the procedure because it is more acceptable to patients, significantly reduces vascular complications, and facilitates faster recovery, among other advantages, compared with PCI through the femoral artery. However, the complicated anatomic relationship between the right radial artery and aorta (greater intersection angles, more blood vessel variations) may result in inconveniences, such as long X-ray exposure times, time-consuming catheter deliveries, catheter placement difficulties, and poor recoil support forces. Moreover, longer times may be required for young physicians or beginners to learn the procedure 1. Therefore, to save time when performing an emergency PCI, a considerable proportion of physicians still choose to utilize the classic femoral artery route. However, it is well known that the femoral artery route significantly increases the risk of bleeding compared with the radial artery route 2. Thus, to avoid the disadvantages of both the right radial artery and the femoral artery routes, we chose to perform emergency PCI via a left femoral artery route and compared this route with the femoral artery route.

## MATERIALS AND METHODS

### Study subjects

This was a prospective study conducted in accordance with guidelines approved by the Ethics Committee of the First Hospital of Hebei Medical University. All subjects signed informed consent forms. The following patients were included in the study: 1). Patients with confirmed diagnoses of acute MI, including ST-segment elevation MI (STEMI) and NSTEMI; 2). patients in whom an emergency PCI was warranted; and 3). patients who volunteered to participate in the study. The following patients were excluded from the study: 1). Patients with cardiovascular complications and 2). patients who were not candidates for emergency PCI.

A total of 206 patients with acute myocardial infarction (AMI) who required emergency PCI and were admitted to our hospital between January 2011 and August 2013 were divided into the following two groups: a group that underwent PCI through the left radial artery (hereafter referred to as the left radial artery group) and a group that underwent PCI through the femoral artery (hereafter referred to as the femoral artery group). Group assignments were made randomly by an experienced surgeon, according to each patient's pulse and hemodynamic status. Patients enrolled in the left radial artery group must have had a positive Allen test. Patients with a negative Allen test were all assigned to the femoral artery group. The general characteristics of the patients are shown in Table 1. The primary endpoint of the study was the recanalization rate, and the secondary endpoint of the study was the one-month survival rate.

### Artery puncture

The arteries of the left radial artery group were punctured using methods described by Seldinger and placed in a radial artery sheath (Terumo 6F). Unfractionated heparin, 3000 IU, and nitroglycerin, 200 µg, were routinely administered. The arteries of the femoral artery group were punctured using the modified Seldinger puncture method and placed in an arterial sheath (Cordis 6F). Unfractionated heparin was routinely given for anticoagulation.

### Catheterization

A Judkins catheter was used for selective coronary angiography. The culprit artery was predetermined by electrocardiographic (ECG) testing. An angiographic catheter (the size of this catheter was 0.5 smaller in the left radial artery group than in the femoral artery group) was first inserted into the non-culprit artery side to explore the anatomy, and then a guiding catheter was inserted directly into the culprit artery side to perform vascular angiography. The times required for the angiographic and guiding catheters to reach the coronary artery opening were recorded. The objective of the surgery was to attempt to open the culprit artery to achieve TIMI flow >2.

### Postoperative treatment

In the left radial artery group, the catheter sheath was removed immediately after the procedure. A general pressure bandage was then applied and loosened layer by layer, if warranted, until it was completely removed after six hours. In the femoral artery group, the catheter sheath was removed under ECG monitoring four hours after the procedure. Then, a pressure bandage was applied, and the patients were immobilized for 12 hours.

### Medication before and after PCI

Before PCI, patients were given anti-platelet treatment, 300 mg of aspirin, 600 mg of clopidogrel, and 20-40 mg of atorvastatin, according to their statuses. Based on their blood pressures and heart rates, some of the patients were also given ester nitrates, such as metoprolol tartrate tablets, or angiotensin converting enzyme inhibitors (ACEIs), such as benazepril. After PCI, all the patients were given 100 mg/d aspirin, 75 mg/d clopidogrel, 20 mg/d atorvastatin and an intravenous drip infusion of tirofiban for 24 hours. Based on their blood pressures and heart rates, some of the patients were also given β-blockers.

### Statistical analysis

SPSS 13.5 Software (Chicago, IL, USA) was used in the statistical analysis. Continuous variables were expressed as the mean ± standard deviation, and the t test was used for comparisons between the groups. Categorical variables were analyzed using an X^2^ test. *p*<0.05 was considered statistically significant.

## RESULTS

### Demographic and general characteristics of the patients

Among the 206 patients enrolled in the study, 108 patients were enrolled in the left radial artery group, including 68 males and 40 females. Their average age was 58±9.6 years. Ninety-eight patients were enrolled in the femoral artery group, including 60 males and 38 females. Their average age was 56±10.7 years. The differences in gender and age between the two groups were not statistically significant.

### Placement times for the angiographic and guiding catheters

There were no significant differences in the times required for the angiographic and guiding catheters to reach their target locations between the two groups (*p*>0.05) (Table 2).

### Procedure success rate and postoperative survival time

In left radial artery group, 5 patients were transitioned to PCI performed through either the femoral artery or the right radial artery due to a left radial artery procedure that was overly time-consuming, 2 patients were transitioned because of spasms that occurred during catheter delivery, and 1 patient was transitioned due to a tortuous subclavian artery that caused procedural difficulties. In the femoral artery group, 2 patients were transitioned to PCI performed through the left radial artery because of difficulties in guiding or manipulating the catheter caused by serious iliac artery tortuousness. There were no significant differences in the procedure success rate or postoperative one-month survival rate between the two groups. (*p*>0.05) (Table 3).

### Postoperative complications

In the left radial artery group, 2 patients developed upper-arm hematomas that fully resolved after treatment, and; 3 patients developed radial artery occlusion, including 2 patients whose arteries were recanalized after local massage and one patient whose artery remained occluded. In the femoral artery group, 1 patient developed a retroperitoneal hematoma (BARC type 3b). This patient was transferred to the medical intensive care unit (MICU) after a blood transfusion but died of a cardiac event after antiplatelet and anticoagulant medications were stopped. Four patients developed pseudoaneurysms, which improved after compression or ultrasound-guided thrombin injections; 6 patients developed subcutaneous blood clots due to injection-induced hematomas; and 4 patients developed vasovagal syncope. There was a significant difference in the incidence of vascular complications between the two groups (*p*<0.05) (Table 4). The femoral artery group had more vascular complications than the left radial artery group, including one severe vascular complication that led to a patient’s death.

## DISCUSSION

During the past decade, there has been a gradual increase in the number of cases in which PCI was performed through the radial artery route, a trend paralleled by an increase in the number of cases in which interventional and direct PCI were performed for STEMI. The results of the performance of emergency PCI through the right radial artery route are not inferior to those of the performance of emergency PCI though the classic femoral artery route 3. According to the results of a study performed by Bertrand in 2010, 89.4% of the transradial PCIs performed worldwide were performed through right radial artery, and only 10.6% were performed through left radial artery 4. In China, many physicians have chosen to use the radial artery route to perform routine PCI; however, for emergency PCI, physicians still prefer using the femoral artery route because the radial artery procedure is associated with many drawbacks, for example, radial artery puncture, difficulties with catheter delivery, or inadequate conduit support. However, the femoral route is also associated with many severe vascular complications. The direction from which the catheter enters the ascending aorta via the left radial artery is similar to that from which it enters the aorta via the femoral artery, and the left subclavian artery shows limited shape/directional variation; thus, choosing the left radial artery route for emergency PCI may enable clinicians to retain the advantages of the radial route and simultaneously avoid the disadvantages of the femoral route and thus should result in satisfactory outcomes. Therefore, in the current study, we assigned 206 patients who recently underwent emergency PCI in our hospital into the indicated two groups (a left radial artery group and a femoral artery group) and compared the two groups with respect to several parameters. We found that emergency PCI through the left radial route can achieve catheter placement results similar to those of the femoral artery route and is as fast and as safe as its counterpart. More importantly, PCI through the left radial route significantly decreased the occurrence of vascular complications, especially complications that were unacceptable to patients. The two groups had similar overall patient prognoses after PCI. This result is consistent with those of the TCT2012 reports, which found that transradial access reduced the risk of bleeding in high-risk acute coronary syndrome (ACS) patients 5. A more recent study has confirmed this conclusion 6.

Vigorous debates about which of the two commonly used emergent PCI methods 7-9, the traditional femoral artery method or the right radial artery method, is superior are still ongoing. Those who support use of the femoral artery method believe that the catheters can provide strong support such that procedures intended to treat more complicated lesions, such as bifurcations, calcified lesions, and tortuous lesions, can be performed. Furthermore, thrombus aspiration can be performed using this method; thus, its success rate is higher than that of the right radial artery method 10. However, the femoral artery method is associated with more postoperative complications than its counterpart, such as pseudoaneurysms, vagal reflex responses, and retroperitoneal hematomas that may require blood transfusion or lead to death in severe cases 11. In addition, ACTs must be measured for 6 hours after surgery, and the arterial sheath must be removed under ECG monitoring. Moreover, the femoral artery must be bandaged under pressure for 24 hours. Since this is uncomfortable, patients do not like to be treated with this method. Regarding the right radial artery method, because the right subclavian artery forms a large angle with the ascending aorta, and significant vascular anatomic variation is possible, it may be difficult to advance the catheter to the right position or provide sufficient conduit support. Thus, the catheter cannot reach the distal vessels when performing thrombus aspiration. For procedures intended to treat complicated cases, such as those characterized by bifurcations, calcified lesions and tortuous lesions, stent is delivery difficult and time-consuming. Ultimately, the success rate of this procedure is lower than that of the classical procedure.

Delivering the guidewire and catheter by the left radial artery method is easier than delivering the guidewire and catheter by the right radial artery method because the left subclavian artery sprouts directly from the aortic arch, and the angle is smaller 12-16. The coaxiality of the catheter and the recoil support force are also better in these procedures than in right radial artery procedures; thus, it is easier to deliver the balloon and stent to distal vessels with lesions. Using this method, the risk of a left internal mammary artery mouth laceration can be reduced in patients who have undergone coronary artery bypass graft surgery (CABG). In addition, the collateral circulation in the left hand is stronger than that in the right hand; thus, choosing to catheterize the left radial artery will not influence the function of the right hand, and the left hand will still have a sufficient blood supply. Furthermore, patients will not need full restrictions, and the arterial sheath can be removed immediately. Thus, this method will be well accepted by patients.

We believe that the left radial artery route is a viable option for emergency PCI, but when difficulties in puncturing the artery or catheter delivery blockages are encountered, a prompt switch to the femoral artery route should be encouraged to save time. It is also worth mentioning that due to the relatively small sample size of this study, our conclusions are still preliminary, and more patients should be recruited in future studies to support the findings of this study.

## AUTHOR CONTRIBUTIONS

Guoqing Qi was responsible for the study design, manuscript writing, analysis of the technique and manuscript revision. Qi Sun was responsible for the manuscript writing, paper revision and table elaboration. Yue Xia and Liye Wei were responsible for the supervision of the study, teaching the technique and manuscript revision.

## Figures and Tables

**Table 1:** The basic clinical characteristics of the patients in the two study groups.

Characteristics	Left radial artery group (n=108)	Femoral artery group (n=98)	*p* value
Age (year)	58±9.6	56±10.7	0.502
Gender (male/female)	68/40	60/38	
Hypertension	82 (75.92%)	70 (71.43%)	
Dyslipidemia	75 (69.44)	65 (66.33%)	
Smoking	32 (29.63%)	35 (35.71%)	
Diabetes	26 (24.07%)	20 (20.41%)	
Number of diseased vessels			
1	60 (55.56%)	57 (58.16%)	
2	34 (31.48%)	26 (26.53%)	
3	14 (12.96%)	15 (15.31%)	

**Table 2:** Angiographic catheter and guiding catheter placement times.

Groups	Left coronary angiographic catheter placement time (seconds)	Right coronary angiographic catheter placement time (seconds)	Left guiding catheter placement time (seconds)	Right guiding catheter placement time (seconds)
Left radial artery group	79.65±26.31	86.43±22.35	81.09±28.17	87.22±15.32
Femoral artery group	76.23±20.41	87.68±18.34	80.46±19.87	85.13±17.08
*p* value	>0.05	>0.05	>0.05	>0.05

**Table 3:** Procedure success rates and one-month postoperative survival rates.

Groups	Success rate (TIMI blood flow >2) (%)	One-month postoperative survival rate (%)
Left radial artery (n=108)	94.44	97.22
Femoral artery (n=98)	95.91	96.94
*p* value	>0.05	>0.05

**Table 4:** Postoperative vascular complications.

Group	Vascular occlusion	Retroperitoneal hematoma	Pseudo-aneurysm	Vasovagal syncope	Local hematoma	Overall vascular event (%)
Left radial (n=108)	3	0	0	0	2	4.63
Femoral artery (n=98)	0	1	4	4	6	15.30

*p*<0.05
